# Hybrid zone analysis confirms cryptic species of banded newt and does not support competitive displacement since secondary contact

**DOI:** 10.1002/ece3.10442

**Published:** 2023-08-30

**Authors:** Konstantinos Kalaentzis, Jan W. Arntzen, Aziz Avcı, Victor van den Berg, Wouter Beukema, James France, Kurtuluş Olgun, Isolde van Riemsdijk, Nazan Üzüm, Manon C. de Visser, Ben Wielstra

**Affiliations:** ^1^ Institute of Biology Leiden, Leiden University Leiden The Netherlands; ^2^ Naturalis Biodiversity Center Leiden The Netherlands; ^3^ Hydrobiological Station of Rhodes, Hellenic Centre for Marine Research Rhodes Greece; ^4^ Department of Biology Aydın Adnan Menderes University Aydın Turkey; ^5^ Reptile, Amphibian and Fish Conservation Netherlands (RAVON) Nijmegen The Netherlands; ^6^ Plant Evolutionary Ecology Institute of Evolution & Ecology, University of Tübingen Tübingen Germany

**Keywords:** geographical cline analysis, KASP genotyping, niche overlap, *Ommatotriton nesterovi*, *Ommatotriton ophryticus*

## Abstract

When two putatively cryptic species meet in nature, hybrid zone analysis can be used to estimate the extent of gene flow between them. Two recently recognized cryptic species of banded newt (genus *Ommatotriton*) are suspected to meet in parapatry in Anatolia, but a formal hybrid zone analysis has never been conducted. We sample populations throughout the range, with a focus on the supposed contact zone, and genotype them for 31 nuclear DNA SNP markers and mtDNA. We determine the degree of genetic admixture, introgression, and niche overlap. We reveal an extremely narrow hybrid zone, suggesting strong selection against hybrids, in line with species status. The hybrid zone does not appear to be positioned at an ecological barrier, and there is significant niche overlap. Therefore, the hybrid zone is best classified as a tension zone, maintained by intrinsic selection against hybrids. While the two banded newt species can evidently backcross, we see negligible introgression and the pattern is symmetric, which we interpret as supporting the fact that the hybrid zone has been practically stationary since its origin (while extensive hybrid zone movement has been suggested in other newt genera in the region). Our study illustrates the use of hybrid zone analysis to test cryptic species status.

## Graphical abstract

Two recently recognized cryptic species of banded newt (genus *Ommatotriton*) are suspected to meet in parapatry in Anatolia, but a formal hybrid zone analysis has never been conducted. We sample populations throughout the range, with a focus on the supposed contact zone, and genotype them for 31 nuclear DNA SNP markers and mtDNA. We reveal an extremely narrow hybrid zone, suggesting strong selection against hybrids, in line with species status.

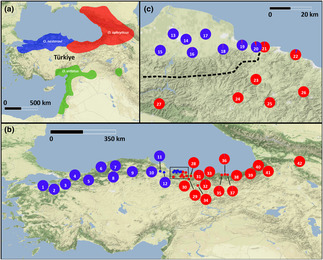

## 1 INTRODUCTION

Cryptic species are genetically distinct populations that, due to their morphological similarity, are currently classified as a single species or have been so up until recently (Bickford et al., 2007; Fiser et al., 2018; Struck et al., 2018). With the genomic revolution, the deluge of molecular data has made it clear that cryptic biodiversity is abundant (Beheregaray & Caccone, 2007; Espindola et al., 2016; Pfenninger & Schwenk, 2007). A proper understanding of cryptic species is crucial in, for example, the fields of ecology, evolution, conservation, medicine, bio‐prospecting, and biological control (Bickford et al., 2007; Espindola et al., 2016). This raises the question: how to best diagnose cryptic species?

Although there are widely varying species concepts, the key factor considered in sexually reproducing taxa, either explicitly or implicitly, is reproductive isolation (Coyne & Orr, 2004). When proposing cryptic species hypotheses, reproductive isolation is often inferred, for example, from genetic distance (Bickford et al., 2007). However, if putative cryptic species meet in parapatry, it is possible to conduct hybrid zone analysis (if they hybridize at all) and determine the degree of selection against hybrids (Barton & Hewitt, 1985; Harrison, 1990; Hewitt, 1988). Hybrid zone analysis allows the most direct test of whether and to what extent barriers to gene flow have evolved between putative cryptic species (Dufresnes et al., 2020).

When hybridizing species have unequal fitness in the region where they establish secondary contact, that is, if one has a competitive advantage over the other, hybrid zone movement would ensue (Buggs, 2007; Wielstra, 2019). Classic hybrid zone theory predicts that moving hybrid zones easily get trapped in troughs of low population density (i.e., ecological barriers) where dispersal is limited (Barton & Hewitt, 1985). However, the rapid accumulation of examples of hybrid zone movement suggests that this phenomenon is likely to be more pervasive than previously appreciated (Wielstra, 2019). The direction of selectively neutral introgression across a hybrid zone provides insight into its dynamic history, as a moving hybrid zone is predicted to leave introgressed alleles in its wake (Buggs, 2007; Currat et al., 2008; Scribner & Avise, 1993; Wielstra, 2019).

Although closely related species are presumably similar in ecology, any ecological differences between them could drive competitive exclusion (Buggs, 2007; Wielstra, 2019). By associating species occurrence data with (a)biotic spatial data layers, potential ecological differences between cryptic species can be exposed (Espindola et al., 2016; Wielstra et al., 2012). Such an analysis could help interpret if the location where a hybrid zone is observed corresponds to a geographical transition in the ecological preferences of the species involved.

The Pleistocene Ice Age has created a hotbed of hybrid zones: while species survive in geographically isolated refugia during relatively cold glacial periods, they can establish secondary contact and form hybrid zones during relatively warm interglacial periods (Hewitt, 2011). As a consequence, most hybrid zones observed today formed after the Last Glacial Maximum subsided, during the current Holocene interglacial. Even though most research initially focused on Europe, a global distribution pattern of Holocene hybrid zones is gradually emerging (Hewitt, 2000, 2004). One area particularly rich in hybrid zones is the “Anatolian suture zone” in western Asia (Bilgin, 2011).

**FIGURE 1:**
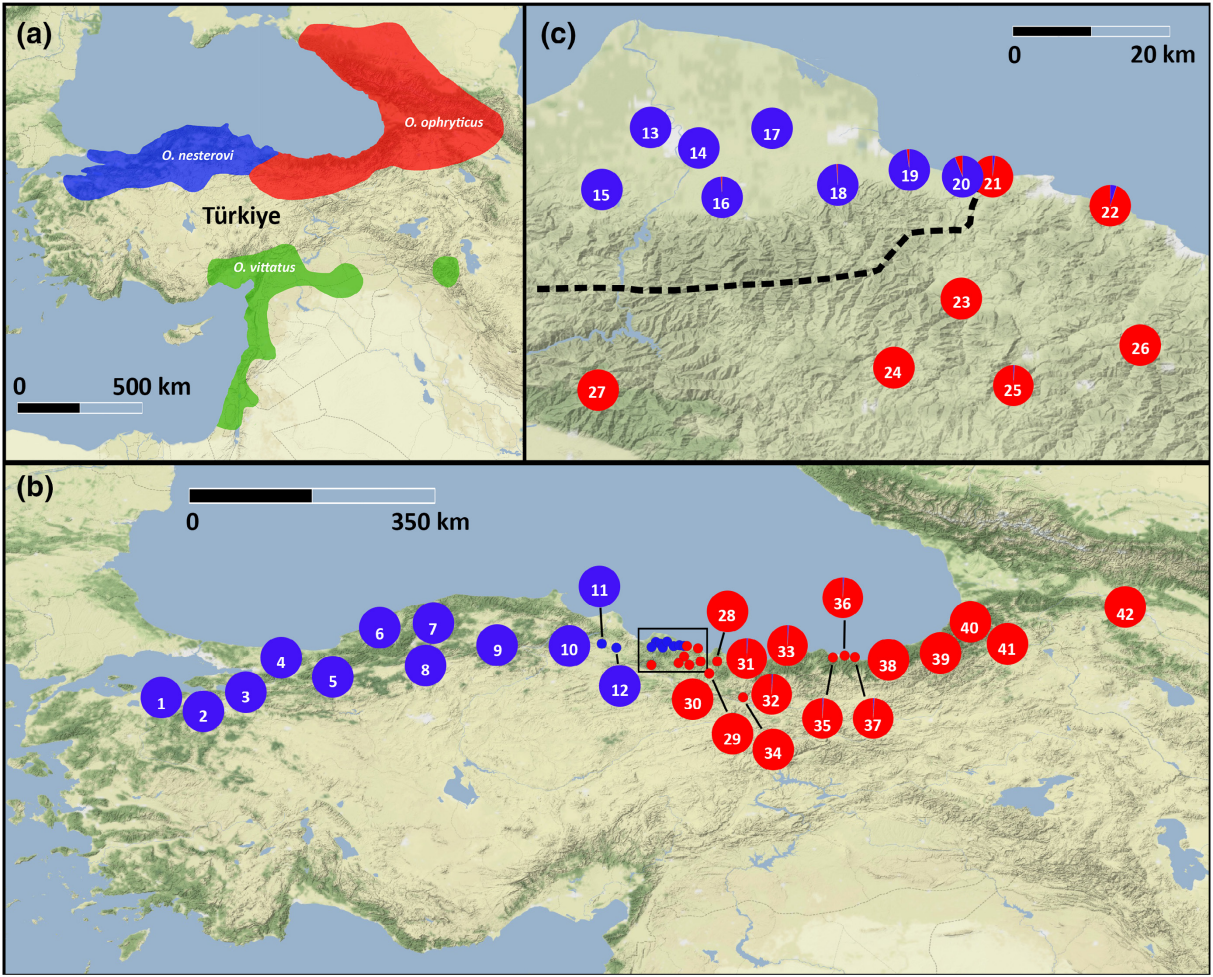
Distribution of the banded newts (genus *Ommatotriton*) in the Near East and sampling scheme. Of the three cryptic banded newt species, one occurs in allopatry in the Levant (a); we focus on the two species presumed to meet in parapatry in northern Türkiye (b, c). The square in panel b reflects the area displayed in panel c. Pie diagrams reflect the nuclear DNA‐based genetic composition of localities (with blue representing *O. nesterovi* and red representing *O. ophryticus*). The isoline in panel c represents the theoretical center of the hybrid zone, based on an interpolation of the nuclear DNA‐based hybrid index observed in sampled localities. Locality numbers correspond to Table 1.

Banded newts (genus *Ommatotriton*) are endemic to the Near East (Figure 1). Up until recently, the banded newt was considered a single species (Borkin et al., 2003), but recent studies propose a treatment as three distinct species (Bülbül & Kutrup, 2013; Frost, 2021; Litvinchuk et al., 2005; Üzüm et al., 2019; van Riemsdijk et al., 2017). These species are characterized by highly diverged mtDNA and nuDNA clades (van Riemsdijk et al., 2017, 2022).

Two of the three banded newt species, *Ommatotriton nesterovi* and *Ommatotriton ophryticus*, occur along the southern shore of the Black Sea. However, their cryptic nature prevented adequate sampling in previous studies—the species were not recognized as such at the time of sampling. Accordingly, a c. 60 km sampling gap between *O. nesterovi* and *O. ophryticus* remained. Curiously, the same two species have been introduced on the opposite side of the Mediterranean Basin in Spain, where they are known to hybridize extensively and form a “hybrid swarm” (van Riemsdijk et al., 2018). Therefore, it is reasonable to assume that the two banded newt species also hybridize where (if) they meet in nature. This putative hybrid zone might well be mobile because, in both other newt genera that occur in the region, extensive hybrid zone movement has been inferred (Wielstra et al., 2013).

We densely sample across the area of interest and genotype over 500 individuals for 32 DNA markers, with the aim of determining the extent of reproductive isolation and introgression between the two species. We also characterize the bioclimatic niches of both species, determine to what extent these niches overlap, and assess if variation in macroclimate may influence the position of the putative hybrid zone. This allows us to test cryptic species status and infer hybrid zone dynamics in banded newts.

## 2 MATERIALS AND METHODS

### 2.1 Sample collection, DNA extraction, mtDNA genotyping

We sampled 512 individuals from 43 localities from the natural range in Türkiye and Georgia and 11 individuals from a locality in Spain, where banded newts have been introduced (Figure 1, Table 1; Table S1). DNA was extracted from tissue samples using the Genomic DNA purification kit (Promega). We obtained sequence data for 658 bp of the mitochondrial DNA marker CO1 for a subset of 200 individuals. For 59 individuals, data were taken from van Riemsdijk et al. (2017, 2018), and for another 141 individuals, data were newly produced following van Riemsdijk et al. (2017). To allocate newly identified haplotypes to species, we constructed a Bayesian phylogeny with MrBayes 3.2.7 (Ronquist et al., 2012), using the settings as in van Riemsdijk et al. (2017); for more details see, Appendix S1.

**TABLE 1:** Sampled banded newt populations.

Code	Locality	Latitude	Longitude	Species	N KASP	Hybrid index	N mtDNA	mtDNA frequency
1	Türkiye: Hurriyet	40.276	28.650	*O. nesterovi*	12	0.00	3	0
2	Türkiye: Avdancik	40.291	29.160	*O. nesterovi*	12	0.00	3	0
3	Türkiye: Mustafali	40.361	29.572	*O. nesterovi*	12	0.00	3	0
4	Türkiye: Levent	40.965	30.433	*O. nesterovi*	12	0.00	3	0
5	Türkiye: Abant Golu	40.612	31.288	*O. nesterovi*	3	0.00	3	0
6	Türkiye: Hacilar	41.497	32.102	*O. nesterovi*	12	0.00	3	0
7	Türkiye: Cal	41.367	32.944	*O. nesterovi*	12	0.00	3	0
8	Türkiye: Basboyunduruk	41.014	32.861	*O. nesterovi*	12	0.00	3	0
9	Türkiye: Cibankoy	41.201	34.037	*O. nesterovi*	12	0.00	3	0
10	Türkiye: Cebeli	41.121	35.330	*O. nesterovi*	12	0.00	3	0
11	Türkiye: Cakiralan	41.182	35.774	*O. nesterovi*	12	0.00	3	0
12	Türkiye: Kethuda	41.116	36.017	*O. nesterovi*	14	0.00	3	0
13	Türkiye: Cumhuriyet	41.213	36.678	*O. nesterovi*	13	0.00	6	0
14	Türkiye: Carsamba	41.186	36.749	*O. nesterovi*	14	0.00	4	0
15	Türkiye: Agcaguney	41.126	36.604	*O. nesterovi*	14	0.00	11	0
16	Türkiye: Tepealti	41.116	36.792	*O. nesterovi*	9	0.01	9	0
17	Türkiye: Sogutlu	41.219	36.863	*O. nesterovi*	14	0.00	7	0
18	Türkiye: Kocaman	41.141	36.974	*O. nesterovi*	14	0.01	9	0
19	Türkiye: Sakarli	41.155	37.069	*O. nesterovi*	6	0.02	6	0
20	Türkiye: Akçay	41.144	37.146	*O. nesterovi*	15	0.06	9	0
21	Türkiye: Unye	41.142	37.196	*O. ophryticus*	15	0.98	9	1
22	Türkiye: Guzeyali	41.105	37.380	*O. ophryticus*	16	0.95	9	1
23	Türkiye: Dizdar	40.969	37.148	*O. ophryticus*	11	1.00	9	1
24	Türkiye: Kizilelma	40.860	37.055	*O. ophryticus*	12	1.00	5	1
25	Türkiye: Bali	40.829	37.235	*O. ophryticus*	14	0.99	5	1
26	Türkiye: Karahamza	40.887	37.423	*O. ophryticus*	13	1.00	5	1
27	Türkiye: Catalan	40.828	36.602	*O. ophryticus*	14	1.00	3	1
28	Türkiye: Kadincik	40.888	37.700	*O. ophryticus*	16	1.00	5	1
29	Türkiye: Cihadiye	40.684	37.567	*O. ophryticus*	16	1.00	3	1
30	Türkiye: Cakirli	40.446	37.483	*O. ophryticus*	14	1.00	3	1
31	Türkiye: Maden	40.949	38.157	*O. ophryticus*	11	0.99	3	1
32	Türkiye: Kockayasi	40.565	38.470	*O. ophryticus*	13	0.99	3	1
33	Türkiye: Espiye	40.955	38.726	*O. ophryticus*	12	0.99	3	1
34	Türkiye: Gokcekent	40.287	38.132	*O. ophryticus*	9	1.00	3	1
35	Türkiye: Yesiltepe Koyu	40.953	39.630	*O. ophryticus*	14	0.99	3	1
36	Türkiye: Yomra	40.983	39.827	*O. ophryticus*	12	0.99	3	1
37	Türkiye: Yesilyali	40.959	39.998	*O. ophryticus*	12	0.99	3	1
38	Türkiye: Denizgoren	40.968	40.380	*O. ophryticus*	4	1.00	3	1
39	Türkiye: Karagol	41.223	41.610	*O. ophryticus*	12	1.00	3	1
40	Türkiye: Ortakoy	41.284	41.913	*O. ophryticus*	12	1.00	3	1
41	Türkiye: Kopruyaka	41.260	42.312	*O. ophryticus*	12	1.00	3	1
42	Georgia: Mtskheta	41.809	44.498	*O. ophryticus*	12	1.00	3	1
I	Spain: Sierra de Busa	42.098	1.647	Admixed	11	0.69	11	1

### 2.2 KASP genotyping

Genotyping for nuclear DNA SNPs was performed using the fluorescence‐based Kompetitive Allele Specific PCR (KASP; Semagn et al., 2014) with the SNPLine platform (LGC Genomics) at the Institute of Biology Leiden (IBL), according to the manufacturer's instructions. Genotype calling was performed using the Kraken software (LGC genomics). Genotypic data can be found in Table S1.

To design nuclear DNA SNP markers, we took sequence data from 59 nuclear DNA markers identified by van Riemsdijk et al. (2022) as being species‐diagnostic for *O. nesterovi* versus *O. ophryticus* (i.e., that did not share alleles in populations sampled away from the putative hybrid zone). Out of these, 53 markers contained SNPs with fixed nucleotide differences for *O. nesterovi* versus *O. ophryticus* (Table S2). SNP primers could be designed for 46 of these markers using the Kraken software and were tested on a subset of 96 individuals. Two SNP markers failed completely, and from the remaining 44, we selected the set of 32 that had the least amount of missing data.

### 2.3 Hardy–Weinberg proportions and marker linkage

Calculations of heterozygote deficit and excess were performed for all 32 nuclear DNA SNP markers using the R package Genepop (Rousset, 2008) to check for a potential departure from Hardy–Weinberg equilibrium (HWE). Because the Bonferroni correction (Rice, 1989) can be overly conservative (Narum, 2006), we chose to account for the independence of tests within markers (P_c_; *p*‐value corrected *for N* = 32). The one marker (n239_var1) that showed a statistically significant heterozygote deficit in four populations was excluded from downstream analyses. We confirmed there was no significant pairwise linkage disequilibrium (LD) between markers in Genepop, using the same *p*‐value correction as above.

### 2.4 Nuclear DNA‐based hybrid index

Based on the remaining 31 nuclear DNA SNP markers, we determined the proportion of diagnostic *O. ophryticus* SNP alleles present in each individual, which amounts to the individual hybrid index, and at each locality, which amounts to the locality hybrid index.

### 2.5 Geographical cline analysis

In order for one‐dimensional geographical clines to be fitted in our sample set, we transformed the two‐dimensional population distribution into a one‐dimensional transect (as in Stankowski et al., 2017; Wielstra, Burke, Butlin, & Arntzen, 2017). To this end, a 0.5 contour of the hybrid index was calculated to represent the theoretical center of the putative hybrid zone. The isoline was interpolated using the population locations and hybrid indices with the R package Akima (Akima et al., 2013). The isoline was drawn in QGIS v.3.14.16 (QGIS Delopment Team, 2016) and trimmed outside the approximate banded newt distribution range (Figure 1c). The distances of localities from the isoline were calculated in QGIS (negative values for *O. nesterovi* and positive values for *O. ophryticus*).

We fitted geographical cline models in R using the package HZAR (Hybrid Zone Analysis using R; Derryberry et al., 2014) for 31 nuclear DNA SNP markers, mtDNA, and the hybrid index, based on the genetic data from the natural range, using scripts available from van Riemsdijk et al. (2019). Different allele frequency and tail shape fits contribute to a total of 15 model variants that can be fitted in HZAR. The calculation of the delta Akaike Information Criterion corrected for small sample size (ΔAICc) for each best model was used to assess the significance of cline model fits (Table S3).

### 2.6 Neutral cline width and effective selection

Expected cline width under neutrality and average effective selection (s*) were calculated based on Barton and Gale (1993). Admixture linkage disequilibrium (*D*′) was calculated from the variance in the hybrid index. Lifetime dispersal distance (*σ*) was estimated, taking into account if individuals were in the pre‐metamorphic or post‐metamorphic stage (because only the latter have had the opportunity to disperse). A mean recombination rate of 0.5 (Baldassarre et al., 2014; Caeiro Dias et al., 2022), a generation time of 5 years (Baskale et al., 2013; Kutrup et al., 2005; Özcan & Üzüm, 2015) and initial secondary contact at 12 Ka (corresponding to the onset of the Holocene; Elmas, 2003) were used as input values.

### 2.7 Estimating heterozygosity and ancestry

Each banded newt individual was given a value of 0, 1, or 2 for each of the 31 nuclear DNA SNP markers, depending on the number of *O. nesterovi* alleles that are present. The genotypic data were analyzed using the R package HIest (Fitzpatrick, 2012), which determines the genetic composition of individuals based on maximum likelihood estimates of ancestry (the fraction of alleles inherited from both parental species) and heterozygosity (the fraction of markers that are heterozygous, i.e., with alleles from both parental species).

### 2.8 Climatic niche overlap

Ordination‐based bioclimatic niche overlap was conducted by first running a principal component analysis (PCA‐env) that was calibrated across a rectangular study area encompassing the Black Sea (Broennimann et al., 2012). To achieve this aim, species occurrence records (Table S4) were combined with a set of seven bioclimatic variables (Fick & Hijmans, 2017), selected based on Pearson's *r* (<.7) scores, that summarize temperature‐ and precipitation extremes and temperature averages relevant to ectotherms such as *Ommatotriton* newts, covering both the reproduction and aestivation seasons. For more details, see Appendix S1. The first two PCA axes were used to create a gridded two‐dimensional environmental space in which species niches were plotted and overlap was measured using Schoener's *D* statistic. Similarity tests were conducted to assess whether the two species were significantly more (dis)similar to each other than expected given the climatic conditions in which they occur. Finally, the PCA scores were translated to geographical space to visualize the extent to which bioclimatic conditions inhabited by both species occur throughout the study area (Peñalver‐Alcázar et al., 2021). For more details, see Appendix S1.

## 3 RESULTS

### 3.1 Hybrid index and mitochondrial DNA

We obtained data for 97.7% of the 31 × 523 = 16,213 potential nuclear DNA SNP genotype calls (Table S1). Calculating the hybrid index based on the 31 nuclear DNA SNP markers reveals that there is little genetic admixture between the two cryptic banded newt species (Figure 1). There is no cytonuclear discordance in the natural range: individuals that are (mostly) *O. nesterovi* or *O. ophryticus* based on the 31 nuclear DNA SNP markers also possess *O. nesterovi* or *O. ophryticus* mitochondrial DNA (Table S4).

### 3.2 Geographical cline analysis

**FIGURE 2:**
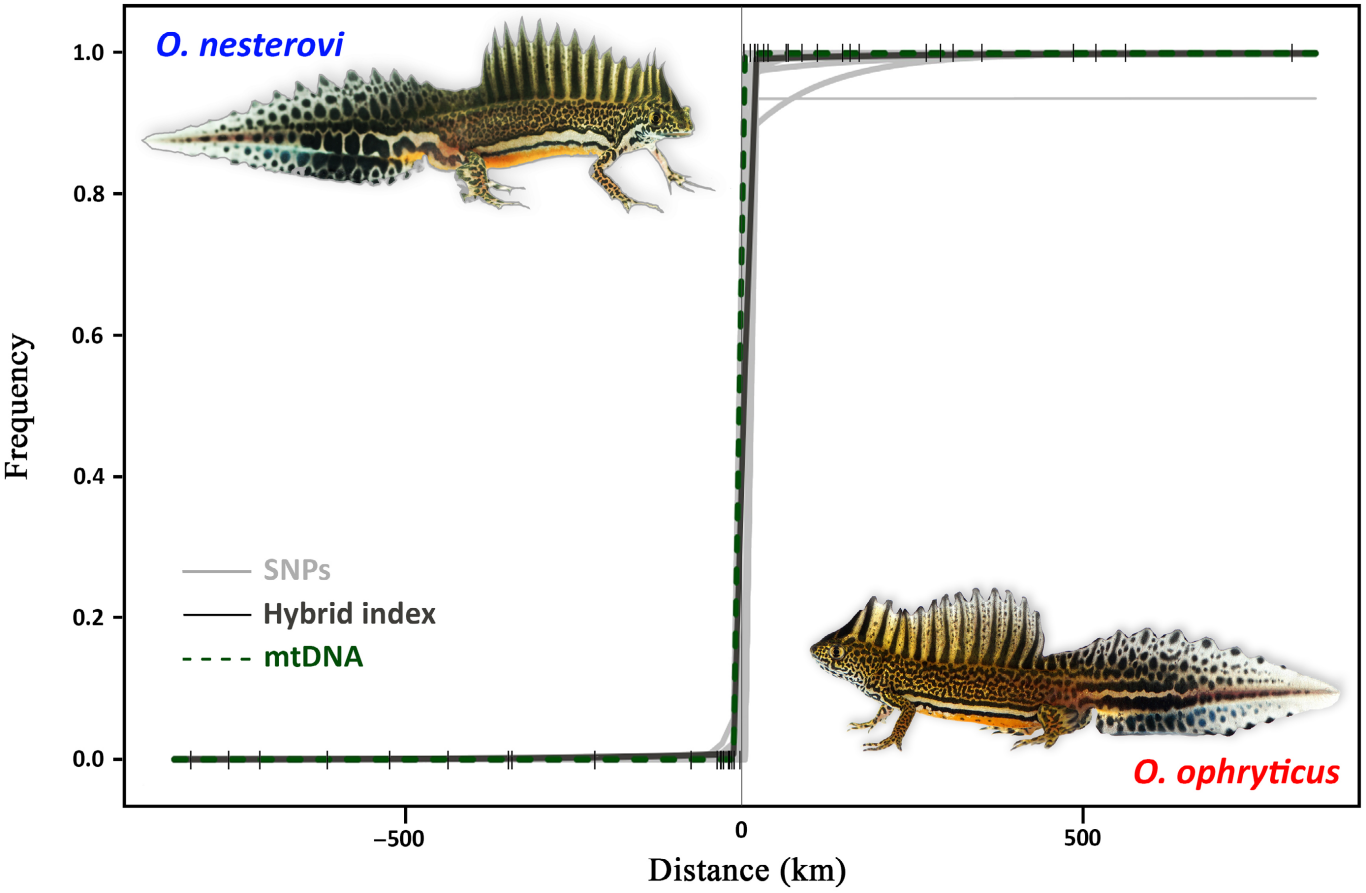
Geographical cline analysis for two banded newt (*Ommatotriton*) species. The vertical line at distance zero reflects the theoretical center of the hybrid zone (the isoline in Figure 1c). Ticks show the position of each population from the cline center. Geographic clines are shown for all individual 31 nuclear DNA SNP markers (gray lines), the hybrid index based on these same nuclear DNA SNP markers (black solid line), and mitochondrial DNA (green dotted line).

The geographical clines for the 31 nuclear markers, mtDNA, and the hybrid index are generally coincident, showing overlapping cline centers, and concordant, showing a steep and narrow transition (Figure 2). The geographical cline based on the hybrid index has a width of 3.37 km (95% CI 0.21–5.33), with the center being placed at −0.18 km (95% CI −2.41 to 2.56) from the theoretical center of the hybrid zone (the isoline in Figure 1c). For only two nuclear DNA markers, the confidence interval for cline width does not overlap with that of the hybrid index, with values being slightly higher, that is, n139_var1 (95% CI 11.23–18.95) and n288 (95% CI 7.42–14.93). For all nuclear DNA markers, the confidence interval for cline center position overlaps with that of the hybrid index (Table S3). The geographical cline of mtDNA has a width of 0.17 km (95% CI 0–5.23), and the center is placed at −0.37 km (95% CI −2.65 to 2.98) from the isoline.

### 3.3 Neutral cline width and effective selection

Lifetime dispersal (*σ*) is estimated at 0.62 km per generation (95% CI 0.45–0.83). The expected cline width under neutrality is 75.81 km (95% CI 55.01–102.04). The average effective selection (s*) is 0.14 (95% CI 0.07–0.24).

### 3.4 Heterozygosity and ancestry

**FIGURE 3:**
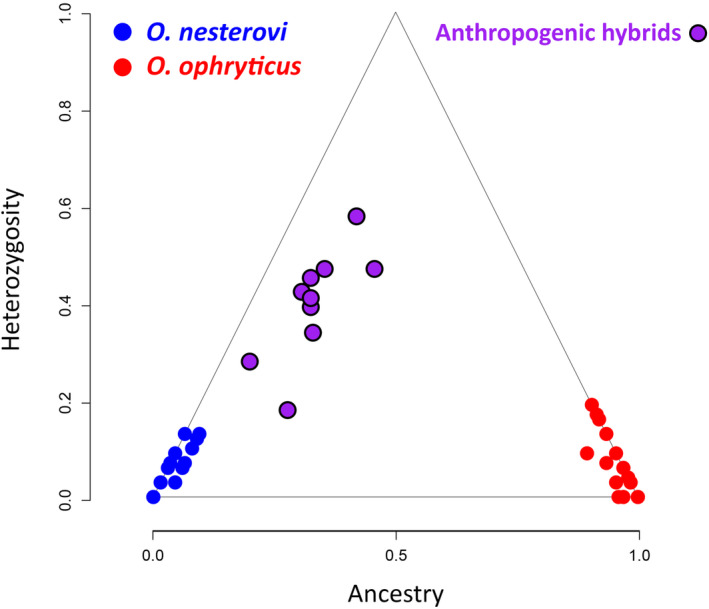
Genetic structure of two banded newt (*Ommatotriton*) species. Plotted is heterozygosity (the fraction of markers that are heterozygous, that is, with alleles from both parental species) versus ancestry (the fraction of alleles inherited from both parental species) of both natural populations and anthropogenic hybrids from the introduced population in Spain, based on 31 nuclear DNA SNP markers. Note that for many individuals, the symbols overlap, particularly in the bottom left and right corners of the plot.

The heterozygosity versus ancestry plot (Figure 3) confirms there is little introgression of note in the natural range (localities 19–22); all individuals are concentrated into two different clusters in the two bottom corners of pure parental genotypes: ancestry values are (close to) 0 or 1 and heterozygosity values are (close to) 0 (Table S1). Individuals sampled from the introduced population in Spain are strongly genetically admixed, with ancestry values ranging from 0.18 to 0.44 and heterozygosity values ranging from 0.2 to 0.6.

### 3.5 Climatic niche overlap

**FIGURE 4:**
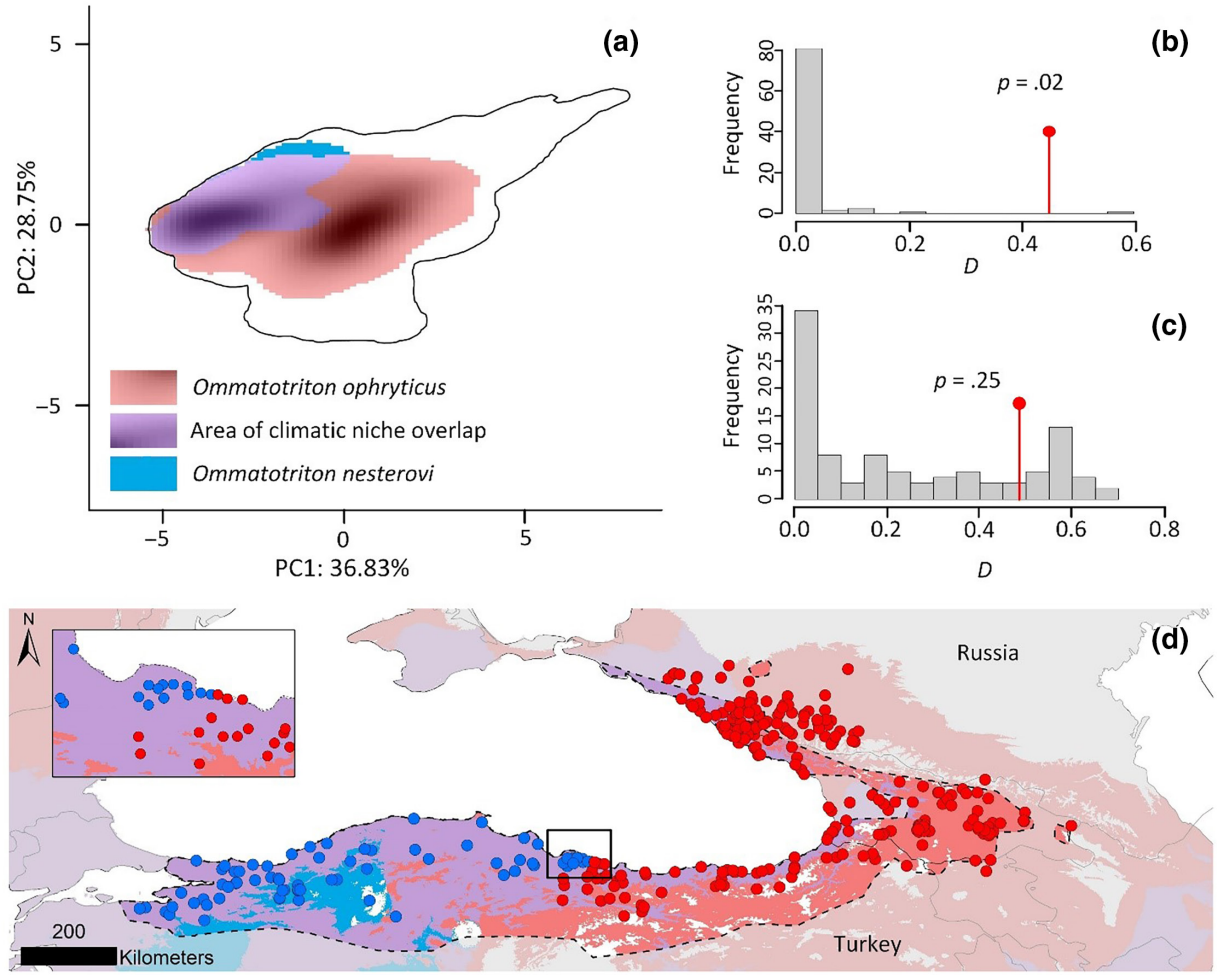
Bioclimatic niche overlap between two banded newt (*Ommatotriton*) species. Two‐dimensional environmental space plot in which species' bioclimatic niches and area of overlap are displayed as kernel density clouds; the black line indicates the border of the study area (a). The niche of *O. nesterovi* is significantly more similar than expected to that of *O. ophryticus* (b), but not the other way around (c). Bioclimatic conditions suitable for both species occur widely (purple areas), including in the hybrid zone (inset); symbols reflect locality data for *O. nesterovi* (blue circles) and *O. ophryticus* (red circles) (d).

The bioclimatic niches of *O. nesterovi* and *O. ophryticus* show considerable overlap in environmental space (Schoener's *D* = 0.48), with the *O. nesterovi* niche largely subsumed in that of *O. ophryticus* (Figure 4a). Indeed, the *O. nesterovi* niche is significantly more similar to that of *O. ophryticus* than expected, given the climatic conditions in which they occur (Figure 4b), but not vice versa (Figure 4c). Translating the climatic niches to geographical space reveals that macroclimate conditions suitable for both species predominate along the southern Black Sea coast (Figure 4d).

## 4 DISCUSSION

### 4.1 Homing in on the banded newt hybrid zone

We confirm the presence of a hybrid zone between two cryptic species of banded newt and pinpoint its location (Figure 1). Previous studies could not determine if the two met in nature, let alone hybridized (Bülbül & Kutrup, 2013; van Riemsdijk et al., 2017, 2022). However, localities from within the previously unsampled region were already known from the literature (Borkin et al., 2003), and an anthropogenic hybrid swarm outside the native range supported hybridization potential (van Riemsdijk et al., 2018). Here we corroborate that genetic admixture does indeed occur in nature. We show that the geographical transition between parental genotypes is abrupt: the distance between the easternmost *O. nesterovi* and the westernmost *O. ophryticus* populations we sampled is merely 4 km as the crow flies (Figure 1).

There is no apparent macroclimatic barrier separating the two cryptic banded newts. Our niche overlap analysis reveals that bioclimatic conditions occupied by both species occur widely along the southern Black Sea coast, including in the hybrid zone (Figure 4). Therefore, the hybrid zone can best be classified as a “tension zone.” Tension zones are maintained through the balancing forces of dispersal of the parental taxa into the hybrid zone and negative selection against their hybrid offspring (Barton & Hewitt, 1985, 1989). We can now use the banded newt hybrid zone to infer the evolutionary independence of the cryptic species (*Is there reduced gene flow?*) and determine if the tell‐tale genomic signature of hybrid zone movement is present (*Is there asymmetric gene flow?*).

### 4.2 Hybrid zone analysis confirms two cryptic banded newt species

Our hybrid zone analysis strongly supports the previously hypothesized species status of *O. nesterovi* and *O. ophryticus* (Bülbül & Kutrup, 2013; Üzüm et al., 2019; van Riemsdijk et al., 2017). Comparison with values for the strength of effective selection (s*) against hybrids between the banded newts (s* = 0.14) and those reported in the literature for recognized amphibian species reveals that s* is 1–2 orders of magnitude higher compared to the hybrid zone between two toad species in France (s* = 0.0017, genus *Bufo*; van Riemsdijk et al., 2019) and two crested newt species in Türkiye (s* = 0.011, genus *Triturus*; Wielstra, Burke, Butlin, Avcı, et al., 2017), whereas it is similar to the classical hybrid zone example of fire‐bellied toads in Poland (s* = 0.17, genus *Bombina*; Szymura & Barton, 1986, 1991).

The strong effective selection against hybrids in the banded newt hybrid zone manifests itself as an extremely narrow (3.37‐km wide) geographical cline for the hybrid index. Based on the lifetime dispersal estimate derived from linkage disequilibrium (0.62 km per generation), it would take less than six generations to cross the hybrid zone, whereas under neutral diffusion, the cline width would be considerably wider (75.81 km). Although the two banded newt species meet, mate, and produce hybrid offspring under natural conditions, they maintain their overall genetic integrity in the face of gene flow. Therefore, we conclude that *O. nesterovi* and *O. ophryticus* are indeed to be considered species. A recent morphological study, guided by recent genetic results, has revealed interspecific differences relating to the “band” that gives the banded newts their name (whether or not it reaches the eye and if it is disrupted by large specks on the tail; for details, see Üzüm et al., 2019). The banded newt case illustrates how hybrid zone analysis can be employed as the ultimate proof for cryptic species status.

### 4.3 The banded newt hybrid zone appears to be static

We do not find a genomic footprint of hybrid zone movement in the banded newt system (Buggs, 2007; Currat et al., 2008; Scribner & Avise, 1993; Wielstra, 2019). In fact, introgression between the two banded newt species is extremely restricted. None of the SNP marker‐specific geographical cline centers are displaced from the hybrid index‐based geographical cline center (Figure 2). While cytonuclear discordance is regularly observed in parapatrically distributed hybridizing species (Toews & Brelsford, 2012)—one could go as far as stating it is the rule rather than the exception—we do not observe mtDNA introgression across the banded newt hybrid zone either (Figure 3).

A genomic footprint of hybrid zone movement can only be registered in the genome if introgression takes place, which requires that F1 hybrids are not an evolutionary dead end so that backcrossing is possible (Mallet, 2005). It could be argued that selection against hybrids in the banded newts is simply too strong for selectively neutral introgression to occur. However, two lines of evidence make this scenario unlikely: (1) we do see evidence of introgression close to the present‐day hybrid zone (Figure 1); and (2) genotyped newts from the invasive hybrid swarm in Spain (van Riemsdijk et al., 2018) are confirmed to be strongly genetically admixed—and *O. ophryticus* mitochondrial DNA is present on a nuclear DNA background that is dominantly *O. nesterovi* (Figure 3).

In light of the observed lack of asymmetric introgression across the banded newt hybrid zone, despite the apparent potential for interspecific introgression, we hypothesize that the banded newt hybrid zone has persisted in the region where it formed upon secondary contact (Pinto et al., 2019). Curiously, two other newt genera along the southern shore of the Black Sea, *Triturus* and *Lissotriton*, do show the pattern of asymmetric introgression expected under hybrid zone movement (Nadachowska & Babik, 2009; Pabijan et al., 2017; Wielstra et al., 2013, 2018; Wielstra & Arntzen, 2016; Wielstra, Burke, Butlin, Avcı, et al., 2017). While the different newt taxa have all been influenced by the same overall geo‐climatic history, their unique responses demonstrate the need for a comparative approach to separate generality from idiosyncrasy. The “Anatolian suture zone” is rich in hybrid zones (Bilgin, 2011) and provides a natural laboratory to conduct such comparative hybrid zone analysis.

## AUTHOR CONTRIBUTIONS

**Konstantinos Kalaentzis:** Conceptualization (equal); data curation (equal); formal analysis (equal); investigation (equal); methodology (equal); resources (equal); software (lead); validation (equal); visualization (lead); writing – original draft (lead); writing – review and editing (equal). **Jan W. Arntzen:** Writing – review and editing (equal). **Aziz Avcı:** Investigation (equal); resources (equal). **Victor van den Berg:** Data curation (supporting); formal analysis (supporting). **Wouter Beukema:** Formal analysis (supporting); resources (supporting); software (supporting); visualization (supporting); writing – review and editing (supporting). **James France:** Data curation (supporting); formal analysis (supporting); methodology (supporting); supervision (supporting); writing – review and editing (supporting). **Kurtuluş Olgun:** Investigation (equal); resources (equal). **Isolde van Riemsdijk:** Data curation (supporting); methodology (supporting); resources (equal); software (supporting); validation (supporting). **Nazan Üzüm:** Investigation (equal); resources (equal). **Manon C. de Visser:** Software (supporting); writing – review and editing (supporting). **Ben Wielstra:** Conceptualization (equal); data curation (equal); formal analysis (equal); funding acquisition (lead); investigation (equal); methodology (equal); project administration (equal); resources (equal); supervision (lead); validation (equal); writing – original draft (equal); writing – review and editing (equal).

## Supporting information


Appendix S1

Table S1

## Data Availability

Raw genotyping data can be found in Table S1. Unique haplotype data are deposited to NCBI Nucleotide Database (see Table S4).
