# Supplementary material for: Hybrid zone analysis confirms cryptic species of banded newt and does not support competitive displacement since secondary contact
Source: Ecol Evol. 2023 Aug 30;13(9):e10442. doi: 10.1002/ece3.10442 (PMC10468612; doi:10.1002/ece3.10442)
Supplement: Supplementary file 1 — Appendix S1 [file ECE3-13-e10442-s001.docx]

**Hybrid zone analysis confirms cryptic species of banded newt and does not support competitive displacement since secondary contact**

Konstantinos Kalaentzis, Jan W. Arntzen, Aziz Avcı, Victor van den Berg, Wouter Beukema, James France, Kurtuluş Olgun, Isolde van Riemsdijk, Nazan Üzüm, Manon C. de Visser, Ben Wielstra

**Supporting Information, Appendix S1: additional methods and results**

*Mitochondrial DNA analysis*

Bayesian phylogenetic reconstruction was performed with MrBayes 3.2.7 (Ronquist et al., 2012). *Lissotriton vulgaris* and *Neurergus strauchii* were added as outgroups (data from Zhang, Papenfuss, Wake, Qu, & Wake, 2008) and two individuals of *Ommatotriton vittatus* were included as well (data from van Riemsdijk et al., 2017). The best fitting models of sequence evolution for each of the three codon partitions (SYM+G, F81, and HKY+G for codon positions 1, 2 and 3) were based on the Bayesian information criterion (Guindon & Gascuel, 2003) in jModelTest 2.1.7 ((Darriba, Taboada, Doallo, & Posada, 2012)). A Markov Chain Monte Carlo search was run for a million generations under default parameters (two runs, four chains, with temperature 0.2), a sampling frequency of 0.001 and a burn-in of 25% of generations. State frequencies were unlinked and rates were set to vary across each of six partitions. Tracer 1.6 (Rambaut, Suchard, & Drummond, 2014) was used to confirm stabilisation within and convergence between runs (all ESS values >200). The resulting phylogeny allowed us to unambiguously assign new haplotypes to either *O. nesterovi* or *O. ophryticus* (Figure S1).


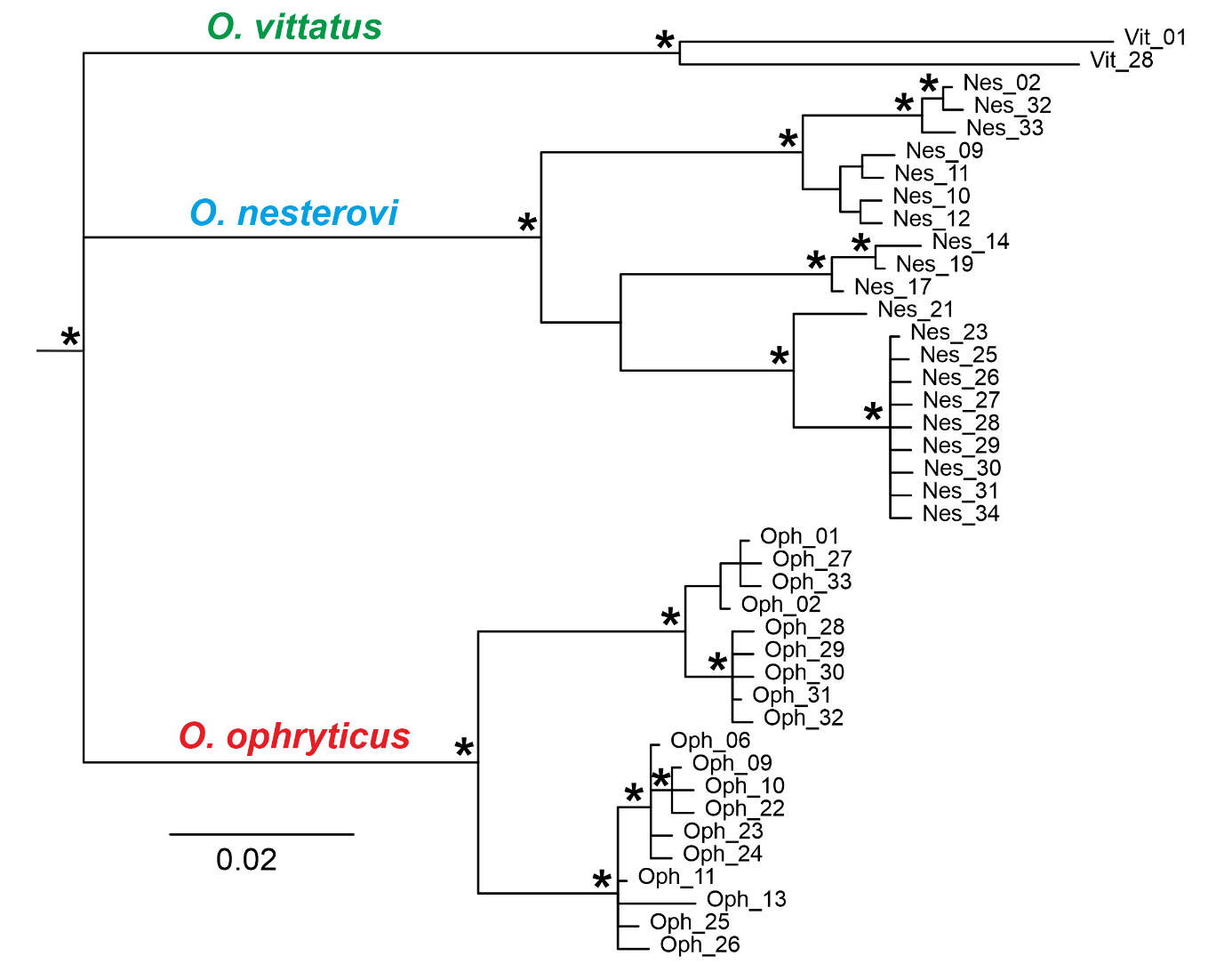


**Figure S1** Mitochondrial DNA phylogenetic tree for banded newts (genus *Ommatotriton*). Asterisks indicate nodes supported with a Bayesian posterior probability ≥ 0.95 (lower values are not shown). Tips are haplotypes with codes corresponding to Table S5. The outgroup is not shown.

*Bioclimatic niche overlap*

To obtain occurrence data for *O. nesterovi* and *O. ophryticus* we combined our newly sampled localities with eight additional unpublished field records and the comprehensive database previously reported by Borkin, Litvinchuk, & Zuiderwijk (2003) and van Riemsdijk et al. (2017) (Table S4). Duplicates at a 1x1 kilometre resolution were removed. This resulted in 68 *O. nesterovi* and 222 *O. ophryticus* localities.

We downloaded the full set of 19 WorldClim bioclimatic variables at approximately 1x1km resolution (Fick & Hijmans, 2017), which we restricted to a rectangular study area centred on the Black Sea and the Caucasus Mountains (Figure 4d). Pairwise Pearson’s *r* scores were calculated to assess the degree of multicollinearity between variable pairs using the *ENMTools* package in R. Decisions on which variable to retain when a pair of variables displayed a Pearson’s *r* higher than 0.7 or lower than -0.7 were made based on expert knowledge of *Ommatotriton* ecology. The final subset used for niche overlap analysis comprised seven variables; Annual Mean Temperature (in °C; bio01), Mean Diurnal Range (in °C, mean of monthly (max temp - min temp); bio02), Temperature Seasonality (in °C, standard deviation ×100; bio04), Min. Temperature of Coldest Month (in °C; bio06), Mean Temperature of Wettest Quarter (in °C; bio08), Mean Temperature of Driest Quarter (in °C; bio09), and Precipitation of Driest Quarter (in mm; bio17). These summarize temperature- and precipitation extremes and temperature averages relevant to ectotherms such as *Ommatotriton* newts, covering both the reproduction and aestivation seasons.

A Principal Component Analysis calibrated on the entire study area (PCA-env; Broennimann et al., 2012) was used as basis for subsequent niche overlap measurements. The two first PCA-axes were subsequently used to generate a 2D gridded representation of environmental space, in which species climatic niches were plotted and overlap was calculated using Schoener’s *D* statistic (Broennimann et al., 2012). This index ranges from 0 (no overlap) to 1 (complete overlap; niche equality). Similarity (or background) tests were performed to compare the overlap between two species to the overlap expected by chance if one or both species was effectively choosing habitat at random from within their broad geographic range. The purpose of this test is to correct for the availability of habitat, and ask whether the observed similarity between species is significantly more (or less) than expected given the available set of environments in the regions in which they occur. Finally, inspired by Peñalver-Alcázar, Jiménez-Valverde, & Aragón (2021), the niches were translated to geographical space to determine the extent to which bioclimatic conditions inhabited by the two species occur throughout the study area.

Climatic niches of the two *Ommatotriton* species in environmental space show considerable overlap (Schoener’s *D* = 0.48), with the *O. nesterovi* niche largely subsumed in that of *O. ophryticus* (Figure 4a). Indeed, the *O. nesterovi* niche is significantly more similar to that of *O. ophryticus* than expected, given the climatic conditions in which they occur, but not true vice versa (Figure 4b, c). The area of overlap (and therefore most of the *O. nesterovi* niche) is characterised by more temperate conditions than the remainder of the *O. ophryticus* niche, including higher annual mean temperatures, lower temperature seasonality, and higher minimum temperatures during the coldest month. The non-overlapping part of the *O. nesterovi* niche is characterised by lower mean temperatures of the wettest quarter than occur within the *O. ophryticus* distribution (unpublished results). Translating the climatic niches to geographical space reveals that climate conditions occupied by both species predominate along the southern Black Sea coast (Figure 4d). Conditions exclusively occupied by *O. nesterovi* coincide with its occurrence in more continental climates of Bolu and Ankara provinces.

**References**

Borkin, L. J., Litvinchuk, S. N., & Zuiderwijk, A. (2003). Bandmolch, Triturus vittatus (Gray, 1835). In *Handbuch der Reptilien und Amphibien Europas* (pp. 555–605).

Broennimann, O., Fitzpatrick, M. C., Pearman, P. B., Petitpierre, B., Pellissier, L., Yoccoz, N. G., … Guisan, A. (2012). Measuring ecological niche overlap from occurrence and spatial environmental data. *Global Ecology and Biogeography*, *21*(4), 481–497. doi: 10.1111/j.1466-8238.2011.00698.x

Darriba, D., Taboada, G. L., Doallo, R., & Posada, D. (2012). JModelTest 2: More models, new heuristics and parallel computing. *Nature Methods*, *9*(8), 772. doi: 10.1038/nmeth.2109

Fick, S. E., & Hijmans, R. J. (2017). WorldClim 2: new 1-km spatial resolution climate surfaces for global land areas. *International Journal of Climatology*, *37*(12), 4302–4315. doi: 10.1002/joc.5086

Guindon, S., & Gascuel, O. (2003). A simple, fast, and accurate algorithm to estimate large phylogenies by naximum likelihood. *Systematic Biology*, *52*(5), 696–704. doi: 10.1080/10635150390235520

Peñalver-Alcázar, M., Jiménez-Valverde, A., & Aragón, P. (2021). Niche differentiation between deeply divergent phylogenetic lineages of an endemic newt: implications for Species Distribution Models. *Zoology*, *144*(June 2020). doi: 10.1016/j.zool.2020.125852

Rambaut, A., Suchard, M. A., & Drummond, A. J. (2014). *Tracer*. Retrieved from www.tree.bio.ed.ac.uk/software/tracer

Ronquist, F., Teslenko, M., van der Mark, P., Ayres, D. L., Darling, A., Hohna, S., … Huelsenbeck, J. P. (2012). MrBayes 3.2: efficient Bayesian phylogenetic inference and model choice across a large model space. *Systematic Biology*, *61*(3), 539–542. Retrieved from 10.1093/sysbio/sys029

van Riemsdijk, I., Arntzen, J. W., Bogaerts, S., Franzen, M., Litvinchuk, S. N., Olgun, K., & Wielstra, B. (2017). The Near East as a cradle of biodiversity: A phylogeography of banded newts (genus Ommatotriton) reveals extensive inter- and intraspecific genetic differentiation. *Molecular Phylogenetics and Evolution*, *114*, 73–81. doi: 10.1016/j.ympev.2017.05.028

Zhang, P., Papenfuss, T. J., Wake, M. H., Qu, L., & Wake, D. B. (2008). Phylogeny and biogeography of the family Salamandridae (Amphibia: Caudata) inferred from complete mitochondrial genomes. *Molecular Phylogenetics and Evolution*, *49*(2), 586–597. doi: 10.1016/j.ympev.2008.08.020
